# MRI-based radiomic features of the urinary bladder wall identify patients with moderate-to-severe international prostate symptom score

**DOI:** 10.1007/s00345-024-05081-3

**Published:** 2024-06-13

**Authors:** Mohammed Shahait, Ruben Usamentiaga, Yubing Tong, Alex Sandberg, David I. Lee, Jayaram K. Udupa, Drew A. Torigian

**Affiliations:** 1 Consultant of Urology, Dubai, UAE; 2 Department of Computer Science and Engineering, University of Oviedo, Gijon, Spain; 3 Medical Image Processing Group, Department of Radiology, University of Pennsylvania, 3710 Hamilton Walk, Goddard Building, 6th Floor, Rm 601W, Philadelphia, PA 19104 USA; 4 Temple Medical School, Temple University, Philadelphia, PA USA; 5 Department of Urology, University of California Irvine, Irvine, CA USA

**Keywords:** Radiomics, MRI, Urinary bladder wall, Machine learning

## Abstract

**Background:**

The International Prostate Symptom Score (IPSS) is a patient-reported measurement to assess the lower urinary tract symptoms of bladder outlet obstruction. Bladder outlet obstruction induces molecular and morphological alterations in the urothelium, suburothelium, detrusor smooth muscle cells, detrusor extracellular matrix, and nerves. We sought to analyze MRI-based radiomics features of the urinary bladder wall and their association with IPSS.

**Method:**

In this retrospective study, 87 patients who had pelvic MRI scans were identified. A biomarker discovery approach based on the optimal biomarker (OBM) method was used to extract features of the bladder wall from MR images, including morphological, intensity-based, and texture-based features, along with clinical variables. Mathematical models were created using subsets of features and evaluated based on their ability to discriminate between low and moderate-to-severe IPSS (less than 8 vs. equal to or greater than 8).

**Results:**

Of the 7,666 features per patient, four highest-ranking optimal features were derived (all texture-based features), which provided a classification accuracy of 0.80 with a sensitivity, specificity, and area under the receiver operating characteristic curve of 0.81, 0.81, and 0.87, respectively.

**Conclusion:**

A highly independent set of urinary bladder wall features derived from MRI scans were able to discriminate between patients with low vs. moderate-to-severe IPSS with accuracy of 80%. Such differences in MRI-based properties of the bladder wall in patients with varying IPSS’s might reflect differences in underlying molecular and morphological alterations that occur in the setting of chronic bladder outlet obstruction.

**Supplementary Information:**

The online version contains supplementary material available at 10.1007/s00345-024-05081-3.

## Introduction

One of the most common conditions that affects older men is benign prostatic hyperplasia (BPH). It is estimated that 50% of men older than 50 years of age show signs of BPH, which is a rapidly growing health concern as the aging population increases globally [1]. Although BPH is not life-threatening, the disease severely impacts the quality of life of those living with the condition due to lower urinary tract symptoms (LUTS). LUTS encompasses a range of both urinary and sexual dysfunctions. The primary reason to seek medical treatment is often prompted by urinary symptoms, characterized by irritative symptoms such as frequency, nocturia, and urgency, as well as obstructive symptoms like diminished flow, dribbling, hesitancy, and irregularity. Several studies indicate that men experiencing LUTS are prone to higher rates of erectile dysfunction, reduced libido, and premature ejaculation [2]. While these symptoms directly correlate with the condition’s pathophysiology, BPH’s negative impact extends beyond urological health to include sleep, mental health, and daily functioning. Therefore, investigation of both treatment strategies and methods for quantifying disease progression has become a key area of exploration. The International Prostate Symptom Score (IPSS) was first developed in 1992 to evaluate symptoms of BPH and guide management. The IPSS consists of a series of seven questions focused on urinary storage and voiding symptoms each on a 0–5 scale ranging from “not at all” to “almost always”. The total score is tallied and categorized as mild (0–7), moderate (8–19), or severe (20–35) [3].

While the exact pathophysiology of LUTS progression remains unclear, it is recognized that prostate enlargement leads to bladder obstruction and eventual remodeling of the bladder wall. Fusco et al. propose a three-stage remodeling sequence of the bladder wall: hypertrophy triggered by mechanical stress, compensated growth, and lastly decompensation marked by smooth muscle loss due to ischemia-reperfusion injury [4]. This study, among others, highlights multiple biological pathways believed to contribute to the remodeling process. *Some studies propose that detrusor wall thickness on ultrasound might aid in the management decision process of LUTS*. However, there is a deficiency in well-defined clinical biomarkers that can effectively monitor bladder progression and its correlation with LUTS. An alternative approach that may prove beneficial is the use of magnetic resonance imaging (MRI) to quantify morphological bladder features. In doing so, MRI would provide a convenient, minimally invasive method of tracking disease progression over time. In this context, we aim to study the correlation between MRI-based bladder radiomics and IPSS.

## Materials and methods

### Study population

The Institutional Review Board at our institution granted approval for this retrospective study, accompanied by a waiver under the Health Insurance Portability and Accountability Act. A total of 140 patients who underwent multiparametric MRI and had simultaneous IPSS assessments during MRI image acquisition were identified. Patients failing to meet quality control standards or had missing clinical parameters were excluded. None of these patients had a history of recurrent UTI, catheter dependence, suspected or known bladder cancer, or bladder diverticula. Figure 1 S represents a visual representation of study participant selection.

The patient cohort was stratified into two groups based on their IPSS scores: the IPSS- mild group (IPSS less than 8) and the IPSS-moderate-to-severe group (IPSS equal to or greater than 8). Among the total patients, 57% exhibited IPSS less than 8, forming the negative group (*N* = 50), while 42% had IPSS equal to or greater than 8, constituting the positive group (*N* = 37).

### Image acquisition and preprocessing

The acquisition of MRI images was conducted for clinical purposes, with our team providing a comprehensive description of the image acquisition process. A summary of patient characteristics is presented in Table 1S. As previously described [5], MRI scans are acquired for clinical evaluation using 1.5 and 3.0 Tesla clinical MRI scanners (Espree model, Siemens Healthcare, Erlangen, Germany). Sagittal T2-weighted turbo spin-echo images of the pelvis are obtained with matrix sizes ranging from 256 × 256 to 512 × 512, comprising 18–40 slices from the aortic bifurcation to the femoral heads. The average slice thickness was 3 mm, with a 3.9 mm gap between slices. Voxel sizes ranged from 0.2 × 0.2 × 3.5 mm^3^ to 0.7 × 0.7 × 4.8 mm^3^.

In order to ensure uniformity across the image dataset, we implemented a series of preprocessing procedures. These steps involved trimming all images to focus solely on the region of interest. Specifically, we delineated both the inner and outer edges of the bladder to distinguish the bladder wall from the lumen and surrounding structures. Additionally, we addressed image inhomogeneity and standardized the intensity levels to facilitate consistent interpretation of tissue regions, as described by Nyul et al. [6]. Subsequently, within each slice of the trimmed and standardized image dataset, the bladder wall was manually identified and delineated. This process was carried out using the CAVASS software system [7], involving collaboration between a medical student and a fellowship- trained urologist. The delineations were further reviewed by a radiologist with expertise in clinical MRI and abdominopelvic imaging for quality assurance.

In Fig. 2S, examples of slices from a single patient are presented, featuring both the standardized MRI slice (top row) and the delineated bladder wall region (bottom row).

Figure 3S illustrates a three-dimensional representation of the delineated bladder wall region. This representation corresponds to a 3D bladder wall volumetric binary image. The bladder wall represents a hollow structure that encloses an empty space within.

### Biomarker discovery approach

Biomarker discovery is a process aimed at identifying and characterizing measurable indicators that may reflect an outcome or state of interest. In this work, the process involves systematic and comprehensive analysis of images to unveil patterns or signatures that can serve as diagnostic indicators. The biomarker discovery method employed in this study follows the optimal biomarker (OBM) method outlined by Tong et al. [8]. Briefly, the procedure begins by extracting an extensive set of quantitative features from the region of interest in the image, specifically the bladder wall region. Subsequently, subsets of features are chosen based on their statistical relevance to the anticipated outcome, and mathematical models are constructed using each selected subset of features. Evaluation of each subset’s performance in predicting the outcome or state of interest guides the identification of the most effective subset of features.

### Feature extraction

The proposed methodology begins by meticulously extracting a diverse set of quantitative features from the designated region of interest within the 3D MRI bladder wall volume. These features are systematically categorized into four distinct groups: morphological, capturing structural characteristics; intensity-based, measuring pixel brightness and histogram properties; texture-based, assessing pixel spatial patterns; and clinical variables, including important factors such as age, prostate size, and body mass index (BMI). This comprehensive feature extraction process ensures a multifaceted representation of the underlying data, incorporating both structural details and relevant clinical information^9^, ^10^. The amalgamation of morphological, intensity-based, texture-based, and clinical variables offers a holistic perspective, enabling a thorough exploration of the 3D MRI bladder wall volume. Further details are discussed in supplementary material 1.

### Optimal feature selection

Feature selection is the process of identifying a set of pertinent features for constructing a classification model. This enhances the model’s accuracy and comprehensibility by reducing the number of features and eliminating unnecessary, redundant, or irrelevant ones. Feature selection encompasses various methods, broadly classified into three main types: filter methods, utilizing statistical measures to assess each feature’s relevance; wrapper methods, employing a learning algorithm to evaluate the performance of different feature subsets; and embedded methods, incorporating the learning of the optimal feature subset as part of the model training process [11]. Existing methods vary in training times and stability, with no singular method consistently outperforming the others [12]. This study adopts a distinctive approach to feature selection, combining the filter method, wrapper method, and embedded technique, as previously described, to identify the most relevant features.

The entire proposed approach is outlined in Fig. 4S. The workflow encompasses the integration of multiparametric magnetic resonance imaging (MRI), expert-driven manual delineation of the region of interest (ROI), and the implementation of the optimal biomarker (OBM) method. Subsequently, optimal feature selection can be utilized to train a model and evaluate patient risk. Further details are discussed in supplementary material 1.

## Results

### Univariate analysis and feature correlation

Table 2S provides a summary of the univariate analysis for features identified as the most effective discriminators (top 10 with the lowest p-values) between positive and negative class groups. All other features within this group pertain to the texture of the bladder wall, characterized by texture descriptors derived from the gray level co-occurrence matrix (GLCM) at various angles (a), distances (d), bins (b), window sizes (w), and features (f). The analysis of features within both intensity-based and morphological categories indicates that none of these features have achieved statistical significance in demonstrating a notable difference between the means of any feature for patients in the positive and negative classes. The overview of morphological features is outlined in Table 3S. These findings imply that features related to the thickness of the bladder wall do not serve as discriminators between positive and negative patients. Table 4S illustrates a summary of the most discriminatory intensity-based features based on the p-value. Similar to morphological features, the p-values suggest that these features lack discriminatory power between positive and negative patients.

### Table 5S summarizes the features in the clinical variable category. Only the mean of the prostate size exhibits a statistically significant difference between the means of positive and negative class patients

In Fig. 5S, a heat map displays the correlation among features, with blue indicating a positive correlation and red indicating negative correlation. The figure reveals substantial correlations among large groups of features, suggesting redundancy in the information they provide. This observation implies that the dimensionality of the feature space can be reduced without sacrificing relevant information. The proposed feature selection approach is specifically designed to address this issue.

### Classification performance

The proposed feature selection approach underwent iterations until convergence, signifying that further tests with new combinations of features did not yield improvement in results. To expedite the execution, certain operations were parallelized by utilizing the GPU and multiprocessing. Each combination of features underwent evaluation through a stratified K-fold cross-validation approach. The feature selection approach yielded an optimal configuration comprising 4 features. Table 6S offers a description of these features utilized in the best classifier. Importantly, all optimally selected features represent textural properties of the bladder wall. Owing to redundancies, the top features identified by the p-value in the different categories of features did not withstand the feature selection process.

The optimal subset of features obtained was assessed through a K-fold cross-validation approach. The dataset was randomly divided into five subsets (5 folds), with each fold preserving a roughly equal class distribution. The model underwent training using four subsets, while the fifth subset was reserved for validation. This cycle repeated five times, ensuring each subset served once for validation. The entire procedure was iterated 100 times, and the outcomes were averaged to improve estimated statistics, resulting in robust and reproducible outcomes. The resulting optimal configuration of features provides a classification accuracy of 0.80 with a sensitivity, specificity, and area under the receiver operating characteristic curve (AUC) of 0.81, 0.81, and 0.87, respectively. The complete performance metrics can be seen in Table 7S. The receiver operating characteristic (ROC) curve is represented in Fig. 6S.

The classifier’s performance can be contrasted with that of a naive classifier, which opts to classify all patients as the majority class. Given the imbalance in the number of patients, with 57% being negative, a naive classifier might choose to label all patients as negative. Such a classifier would yield an accuracy of 0.57 and a balanced accuracy (average of sensitivity and specificity) of 0.5. A classifier constructed using a subset of features that produces comparable metrics may be considered insignificant in terms of its discrimination capabilities.

Table 8S presents a comparison of performance using various subsets of features. Age, a typical clinical feature for distinguishing different medical outcomes, exhibits limited discriminatory power, yielding a classification accuracy of 0.61. Even less favorable results are observed when using BMI. While the combination of the three clinical variables marginally enhances the results, they still fall short of those achieved using the optimal feature subset.

Alternative feature selection methods from the literature were explored, encompassing Minimum Redundancy Maximum Relevance (MRMR), Chi-square, analysis of variance (ANOVA), and Kruskal-Wallis [13]. Each method yields a distinct set of features. Across all instances, when opting for the top ten features, the resultant classification accuracy ranges between 60% and 70%, significantly inferior to the accuracy achieved with the proposed feature selection approach.

## Discussion

BPH is acknowledged as a histologic diagnosis characterized by an augmentation of stromal and glandular epithelial cells in the prostate’s transition zone [14]. Imaging-based parameters, such as prostate size, detrusor muscle thickness, and post-void residual, can be used to aid in establishing the diagnoses of BPH. They also guide subsequent management. In this study we analyzed 7,666 MRI-derived radiomic features of the bladder wall and identified a subset of features that correlates with IPSS in a cohort of men who underwent MRI. All of these discriminatory features were discovered to be features related to texture only, and not morphological, intensity-based, and clinical variable features. The optimal configuration of features provides a classification accuracy of 0.80 with a sensitivity, specificity, and AUC of 0.81, 0.81, and 0.87, respectively.

In population-based studies, it was observed that severe LUTS, defined by an IPSS of 7 or higher, are linked to the onset of acute urinary retention (AUR), a condition indicating the progression of BPH. The incidence of AUR increased from 6.8 episodes per 1,000 patient-years in the overall group to as high as 34.7 episodes among men over 70 years old with moderate-to-severe LUTS. While several studies showed the utility of IPSS in establishing the diagnosis of BPH, choosing treatment modalities, and following-up BPH patients, there are limited data on the association of IPSS severity with bladder contractility and remodeling stage. While LUTS in BPH have been associated with overall reduction in detrusor wall vascularity and oxygenated hemoglobin establishing ischemia, a recent systematic review showed that no individual biomarkers are strongly associated with LUTS. Moreover, this study highlighted the existing gap in this field and suggested considering “fingerprints” of multiple molecules to understand, diagnose, and treat LUTS. There have been a several attempts at noninvasive approaches to evaluate and treat BPH. In particular, urodynamics has been shown to accurately diagnose bladder outlet obstruction and predict surgical outcomes. However, various urodynamics measurements lack a firm correlation with underlying pathologies such as bladder wall ischemia and remodeling, or with the clinical symptoms [15–17]. Few studies have utilized pelvic imaging as a tool to guide BPH symptom management. Guneyli et al. investigated MRI based prostate parameters in 61 patients and correlated with IPSS and found that transitional zone volume was the only statistically significant parameter [18]. The authors of this study called for further exploration of MRI to understand the diverse phenotypes of BPH. We attempted to further study BPH using textural analysis, not only for correlative analysis but also for predictiveassessment as well.

Textural analysis using MRI is a relatively recent tool that has not been extensively researched for the study of BPH. Recent studies have demonstrated its effectiveness in detecting and stratifying the risk of prostate cancer by identifying specific texture parameters [19]. Integrating artificial intelligence is a crucial advancement in enhancing the viability of textural analysis in the management of urological disorders. Although the use of AI in BPH management has been expanding, the research still lacks robust generalizability [20]. The current study delves deeper into the application of AI and textural-based analysis in assessing bladder radiomic features.

In the present study, several complex bladder wall textural features, particularly those derived from the Gray Level Co-occurrence Matrix (GLCM), could discriminate between the two groups. GLCM features, which capture second-order statistical relationships of pixel gray levels, are hypothesized to reflect underlying pathomorphological textures that are not visually apparent. This approach revealed a noteworthy correlation between specific bladder muscle textural features and the IPSS, indicating that radiomics can offer unique insights into LUTS complexity. However, our study found no significant correlation between IPSS and conventional morphological or intensity-based features, such as bladder muscle thickness or pixel intensities. This finding highlights the necessity of exploring beyond traditional measurements to include intricate textural properties that may unveil deeper pathophysiological changes. The interrelationship of variables in complex datasets was evident in the high correlation among certain features, suggesting that they capture overlapping information. This understanding is crucial in feature selection, influencing the importance attributed to individual features and leading to the preference of one feature over others due to redundancy concerns. This approach is consistent with the principle of model simplicity, where features with high correlation might not add significant new insights.

A recent study found a unique radiomic phenotype, which connects differences in muscle tissue found through ultrasound radiomics to several health conditions in older people, including hearing loss, stroke, heart attack, dementia, frailty, and falls [21, 22]. This study suggested that the observed muscle dysfunction in radiomic imaging might be linked to mitochondrial dysfunction, which plays a crucial role in numerous cellular energy processes. Additionally, our research team has found a relationship between radiomic features of the levator ani muscle and postoperative continence outcomes following radical prostatectomy [5]. This serves as another instance demonstrating the wider utilization of radiomic analyses in their connection with urologic clinical outcomes.

There are several limitations to our study, which include but are not limited to a small sample size, and a retrospective study design. Moreover, due to the exploratory nature of the study, a formal power analysis could not be carried out. We utilized available sagittal small field T2-weighted fast spin echo MR images to segment the entire bladder wall. These images, which were acquired in routine clinical practice for screening and evaluation of prostate cancer patients, were not obtained specifically to study the bladder wall, such that it was not feasible to segment the detrusor muscle alone. Therefore, the baseline characteristics of this cohort might differ from those of indexed BPH patients. Furthermore, urodynamic measures such as flow rate, peak flow, detrusor pressure, and post void residual were not incorporated in our model, as these measures were not part of the clinical workup for this patient cohort. Future efforts may consider incorporation of such measures into our prediction model.

It is important to note that our approach in this study has yet to be externally validated. Despite these limitations, the results presented show potential and warrant further investigation in larger, prospective studies.

In conclusion, this study showcased that an independent set of features derived from MRI scans of the urinary bladder can effectively differentiate individuals with low and moderate-to-severe IPSS, achieving an accuracy of 80%. The detected variations in MRI- based characteristics of the bladder wall among patients with distinct IPSS levels suggest potential underlying molecular and morphological changes linked to chronic bladder outlet obstruction. Further investigations examining MRI scans of bladder features can contribute to a deeper understanding of these associations and ultimately help advance the clinical management of BPH.

## Electronic supplementary material

Below is the link to the electronic supplementary material.

Supplementary Material 1

Supplementary Material 2

Supplementary Material 3

Supplementary Material 4

Supplementary Material 5

Supplementary Material 6

Supplementary Material 7

Supplementary Material 8

Supplementary Material 9

Supplementary Material 10

Supplementary Material 11

Supplementary Material 12

Supplementary Material 13

Supplementary Material 14

Supplementary Material 15

## Data Availability

Data are available upon request from the reviewer.
