# Supplementary material for: MRI-based radiomic features of the urinary bladder wall identify patients with moderate-to-severe international prostate symptom score
Source: World J Urol. 2024 Jun 13;42(1):375. doi: 10.1007/s00345-024-05081-3 (PMC11176201; doi:10.1007/s00345-024-05081-3)
Supplement: Supplementary file 1 — Supplementary Material 1 [file 345_2024_5081_MOESM1_ESM.docx]

**Supplementary material 1:**

### *Feature extraction*

Morphological features encapsulate the characteristics related to the shape, size, and spatial arrangement of the region of interest within the volume. Global attributes, such as volume and surface area, provide an overarching perspective. For the bladder wall, distinctive features pertaining to thickness are extracted. The process for determining thickness from the 3D volume commences with identifying the surface points of the binary volume—those points where the binary value transitions from 0 (background) to 1 (object) or vice versa. Subsequently, thickness is measured at each surface point by calculating the distance from each of these points to the nearest point on the opposite side of the object. The representation of thickness involves the use of averages and other statistical measures derived from these distances. These statistical measures reflect thickness centrality and variability, such as median, mean, mode, geometric mean, range, standard deviation, interquartile range (IQR), and percentiles. Furthermore, these thickness-related features are calculated in relation to the total volume of the bladder wall, enriching the contextual understanding of the structural characteristics. Overall, 33 morphological features were calculated for each patient.

Intensity-based features comprise statistical measures characterizing the intensity or brightness values associated with voxels within the bladder wall. These encompass a diverse set of metrics, including the mean, median, standard deviation, mode, maximum, minimum, quartiles, moments, skewness, kurtosis, and peak height. In essence, these features offer a comprehensive insight into the distribution and variation of pixel intensities within the bladder wall region. To quantify these characteristics for each patient, a total of 14 intensity features were systematically extracted. This extensive set of metrics captures the central tendency and dispersion of voxel intensities.

Texture-based features serve the purpose of quantifying the perceived texture or heterogeneity within the bladder wall. This analysis incorporates two distinct types of texture descriptors: local binary pattern (LBP) and gray level co-occurrence matrix (GLCM) [9, 10]. From the GLCM, a multitude of features is derived, considering various factors such as different angles (45, 90, 135, 180, and 360 degrees), distances (1, 3), bins

(5, 10, 20), window sizes (1, 2, 3), and features (1 to 6). This comprehensive approach ensures a detailed exploration of textural characteristics under diverse settings. Simultaneously, LBP contributes to the feature set with considerations for different values of radius (1 and 3) and samples (8 and 12). In total, an extensive array of 7,616 texture features is extracted from each patient (Supplementary Table 1). This broad spectrum of texture descriptors captures the nuanced aspects of texture heterogeneity.

Incorporating additional factors that could potentially influence the outcome, we have considered supplementary features such as age, prostate volume, and BMI. These demographic and physiological variables are integrated with the previously elucidated image-derived features. The amalgamation of these diverse feature sets aims to create a comprehensive analytical framework that captures both the radiological nuances and the broader contextual factors associated with age, prostate volume, and BMI. In this study, feature transformation techniques were intentionally omitted to avoid potential loss of the original measurements’ meaning. Transformed features can be challenging to interpret and may lose their physical relevance. To preserve the intrinsic meaning of the features for diagnostic and therapeutic purposes, only those with direct interpretations in terms of shape, intensity, or texture were employed in the analysis. This decision ensures that the extracted features retain their meaningful context and contribute to a more clinically interpretable and applicable radiomic analysis.

Supplementary Table 1: Summary of the features.

| Category | Number of features | Description |
| --- | --- | --- |
| Morphological | 33 | Volume, surface area, thickness statistics, *. . .* |
| Intensity | 14 | Max, min, std, mean, median, mode, moments, *. . .* |
| Texture | 7,616 | Features from local binary patterns and co-occurrence matrix |
| Clinical | 3 | Age, prostate volume, and body mass index |
| Total | 7,666 |  |

### *Optimal feature selection*

In the proposed approach, sets of features are chosen based on two criteria: (i) minimal correlation among all features, and (ii) maximum discriminatory power between the two classes. These criteria are assessed on a randomly selected training subset comprising 60% of the samples. This stochastic methodology combines statistical significance and randomness to identify robust feature groups, proving particularly effective in managing a substantial number of features within a complex search space. Each feature group undergoes evaluation using a classifier through a repeated (100 times) stratified K-Fold (5-fold) cross-validator. This process is iterated to yield the optimal candidate feature group. Subsequently, all conceivable combinations of features within this group, ranging from 1 to N features, are evaluated using the same approach. The proposed approach aims to yield stable and reproducible outcomes with a limited number of samples.

Stratified K-Fold cross-validation is used to ensure that each fold or subset of the dataset maintains the same class distribution as the original dataset. This is particularly important when dealing with imbalanced datasets, where the number of instances in each class is significantly different. In a stratified K-Fold cross-validation, the dataset is divided into K subsets, or folds, while preserving the proportion of each class in every subset. This ensures that each fold is a representative sample of the overall class distribution. The model is trained and validated multiple times, each time using a different fold as the validation set and the remaining folds for training. By repeating this process K times and averaging the results, the evaluation of the model becomes more robust and less sensitive to the initial random partitioning of the data. In addition to addressing class distribution imbalances, stratified K-Fold cross-validation is robust for preventing overfitting. By subjecting the model to diverse subsets of the data during both training and validation, it becomes less prone to overfitting to a specific subset. Consequently, the proposed approach not only mitigates class distribution imbalances but also aids in preventing overfitting by facilitating a comprehensive and representative evaluation of the model’s performance across various data subsets.
