# Supplementary material for: MRI-based radiomic features of the urinary bladder wall identify patients with moderate-to-severe international prostate symptom score
Source: World J Urol. 2024 Jun 13;42(1):375. doi: 10.1007/s00345-024-05081-3 (PMC11176201; doi:10.1007/s00345-024-05081-3)
Supplement: Supplementary file 2 — Supplementary Material 2 [file 345_2024_5081_MOESM2_ESM.docx]

Table 1: Summary of patient cohort characteristics showing median and interquartile range (IQR).

| Variable |  | Overall N=87 |
| --- | --- | --- |
| Age (years) | Median | 63.30 |
|  | IQR | 58.11-70.48 |
| BMI (kg/m^2^) | Median | 27.57 |
|  | IQR | 25.09-31.11 |
| Prostate size (ml) | Median | 40.00 |
|  | IQR | 29.00-53.00 |
| IPSS | *<*8 | 50 |
|  | *≥*8 | 37 |

BMI: Body Mass Index, IPSS: International Prostate Symptom Score
