# Supplementary material for: MRI-based radiomic features of the urinary bladder wall identify patients with moderate-to-severe international prostate symptom score
Source: World J Urol. 2024 Jun 13;42(1):375. doi: 10.1007/s00345-024-05081-3 (PMC11176201; doi:10.1007/s00345-024-05081-3)
Supplement: Supplementary file 3 — Supplementary Material 3 [file 345_2024_5081_MOESM3_ESM.docx]

Table 2: Analysis of the most effective individual features through univariate analysis, with features sorted by p-value (top 10). The table presents the mean values for each feature concerning positive and negative patients, along with the variability of the means and the corresponding p-values.

| Feature | Mean (pos.) | Mean (neg.) | Std (pos.) | Std (neg.) | p-value |
| --- | --- | --- | --- | --- | --- |
| GLCM mode a45 d1 b5 w3 f1 | 43191.59 | 60961.48 | 29039.16 | 15697.11 | 0.0004 |
| GLCM mode a90 d3 b5 w3 f1 | 43098.70 | 60854.46 | 29158.44 | 16047.50 | 0.0005 |
| GLCM mode a180 d3 b5 w3 f1 | 43088.30 | 60826.08 | 29171.28 | 16132.49 | 0.0005 |
| GLCM mode a135 d3 b5 w3 f1 | 43196.03 | 60816.28 | 29032.50 | 16165.75 | 0.0005 |
| GLCM mode a360 d1 b5 w3 f4 | 1364.05 | 205.84 | 2085.92 | 922.76 | 0.0008 |
| GLCM mode a135 d1 b5 w3 f1 | 46363.76 | 61962.66 | 28057.49 | 14283.90 | 0.0011 |
| GLCM mode a90 d1 b5 w2 f1 | 45306.30 | 60938.10 | 27868.41 | 15747.51 | 0.0013 |
| GLCM mode a360 d3 b5 w3 f4 | 2832.43 | 677.74 | 4016.43 | 2008.07 | 0.0015 |
| GLCM mode a45 d3 b5 w3 f1 | 43269.78 | 59674.22 | 28937.34 | 17768.51 | 0.0016 |
| GLCM mode a360 d3 b5 w2 f4 | 2712.73 | 617.66 | 3892.60 | 2133.88 | 0.0019 |
