# Supplementary material for: MRI-based radiomic features of the urinary bladder wall identify patients with moderate-to-severe international prostate symptom score
Source: World J Urol. 2024 Jun 13;42(1):375. doi: 10.1007/s00345-024-05081-3 (PMC11176201; doi:10.1007/s00345-024-05081-3)
Supplement: Supplementary file 4 — Supplementary Material 4 [file 345_2024_5081_MOESM4_ESM.docx]

Table 3: Univariate analysis of features within the morphological category, organized by p-value (top 10).

| Feature | Mean (pos.) | Mean (neg.) | Std (pos.) | Std (neg.) | p-value |
| --- | --- | --- | --- | --- | --- |
| Volume (mm^3^) | 47627.46 | 41223.91 | 17465.53 | 17678.05 | 0.0968 |
| Relative thickness IQR range | 0.00 | 0.00 | 0.00 | 0.00 | 0.2225 |
| Thickness percentile 5 (mm) | 2.54 | 2.20 | 1.21 | 1.53 | 0.2617 |
| Relative thickness STD | 0.00 | 0.00 | 0.00 | 0.00 | 0.3311 |
| Thickness percentile 1 (mm) | 1.55 | 1.40 | 0.66 | 0.83 | 0.3714 |
| Thickness mode (mm) | 5.46 | 4.82 | 3.39 | 3.48 | 0.3907 |
| Thickness percentile 10 (mm) | 3.21 | 2.88 | 1.65 | 1.87 | 0.3915 |
| Relative thickness percentile 99 | 0.00 | 0.00 | 0.00 | 0.00 | 0.4132 |
| Relative thickness percentile 95 | 0.00 | 0.00 | 0.00 | 0.00 | 0.4535 |
| Relative thickness percentile 75 | 0.00 | 0.00 | 0.00 | 0.00 | 0.4667 |
