# Supplementary material for: MRI-based radiomic features of the urinary bladder wall identify patients with moderate-to-severe international prostate symptom score
Source: World J Urol. 2024 Jun 13;42(1):375. doi: 10.1007/s00345-024-05081-3 (PMC11176201; doi:10.1007/s00345-024-05081-3)
Supplement: Supplementary file 5 — Supplementary Material 5 [file 345_2024_5081_MOESM5_ESM.docx]

Table 4: Univariate analysis of features in the category intensity-based sorted by p-value (top 10). Intensity values are represented in standardized pixel values (no unit) obtained after performing MRI intensity standardization.

| Feature | Mean (pos.) | Mean (neg.) | Std (pos.) | Std (neg.) | p-value |
| --- | --- | --- | --- | --- | --- |
| Minimum | 47.22 | 23.34 | 70.78 | 51.46 | 0.0718 |
| Second moment | 107.60 | 129.20 | 44.28 | 79.88 | 0.1416 |
| Skewness | 32.06 | 40.47 | 16.21 | 39.62 | 0.2272 |
| Third moment | 45262.17 | 120601.14 | 53911.40 | 407029.59 | 0.2671 |
| IQR low Intensity | 384.30 | 353.64 | 120.15 | 137.21 | 0.2808 |
| Kurtosis | 3732.02 | 6453.38 | 2381.96 | 15424.52 | 0.2911 |
| Maximum | 2245.49 | 2581.08 | 829.68 | 1821.22 | 0.3001 |
| Fourth moment | 66739928.09 | 607150193.98 | 106981090.71 | 3523770337.99 | 0.3544 |
| Median | 500.16 | 469.64 | 146.33 | 185.84 | 0.4107 |
| Average | 543.52 | 518.40 | 146.24 | 184.27 | 0.4955 |
