# Supplementary material for: MRI-based radiomic features of the urinary bladder wall identify patients with moderate-to-severe international prostate symptom score
Source: World J Urol. 2024 Jun 13;42(1):375. doi: 10.1007/s00345-024-05081-3 (PMC11176201; doi:10.1007/s00345-024-05081-3)
Supplement: Supplementary file 6 — Supplementary Material 6 [file 345_2024_5081_MOESM6_ESM.docx]

Table 5: Univariate analysis of features in the clinical variable category sorted by p-value.

| Feature | Mean (pos.) | Mean (neg.) | Std (pos.) | Std (neg.) | p-value |
| --- | --- | --- | --- | --- | --- |
| Prostate size (ml) | 56.82 | 41.58 | 33.16 | 25.64 | 0.0177 |
| BMI (kg/m^2^) | 29.45 | 28.67 | 6.29 | 4.97 | 0.5201 |
| Age (years) | 64.01 | 63.62 | 8.00 | 7.82 | 0.8225 |
