# Supplementary material for: MRI-based radiomic features of the urinary bladder wall identify patients with moderate-to-severe international prostate symptom score
Source: World J Urol. 2024 Jun 13;42(1):375. doi: 10.1007/s00345-024-05081-3 (PMC11176201; doi:10.1007/s00345-024-05081-3)
Supplement: Supplementary file 7 — Supplementary Material 7 [file 345_2024_5081_MOESM7_ESM.docx]

Clinical variables

3. Feature selection Modified OBM model

2. Image preprocessing

Trimming, Standardization, ROI specification

4. Model building

Classification

Patient
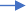
 Patient risk

1. Image acquisition

mpMRI Scanning

Supplementary Figure 4: Workflow of the proposed approach, integrating multiparametric magnetic resonance imaging (MRI), delineation of the region of interest (ROI), and the optimal biomarker (OBM) method.
