# Supplementary material for: MRI-based radiomic features of the urinary bladder wall identify patients with moderate-to-severe international prostate symptom score
Source: World J Urol. 2024 Jun 13;42(1):375. doi: 10.1007/s00345-024-05081-3 (PMC11176201; doi:10.1007/s00345-024-05081-3)
Supplement: Supplementary file 8 — Supplementary Material 8 [file 345_2024_5081_MOESM8_ESM.docx]

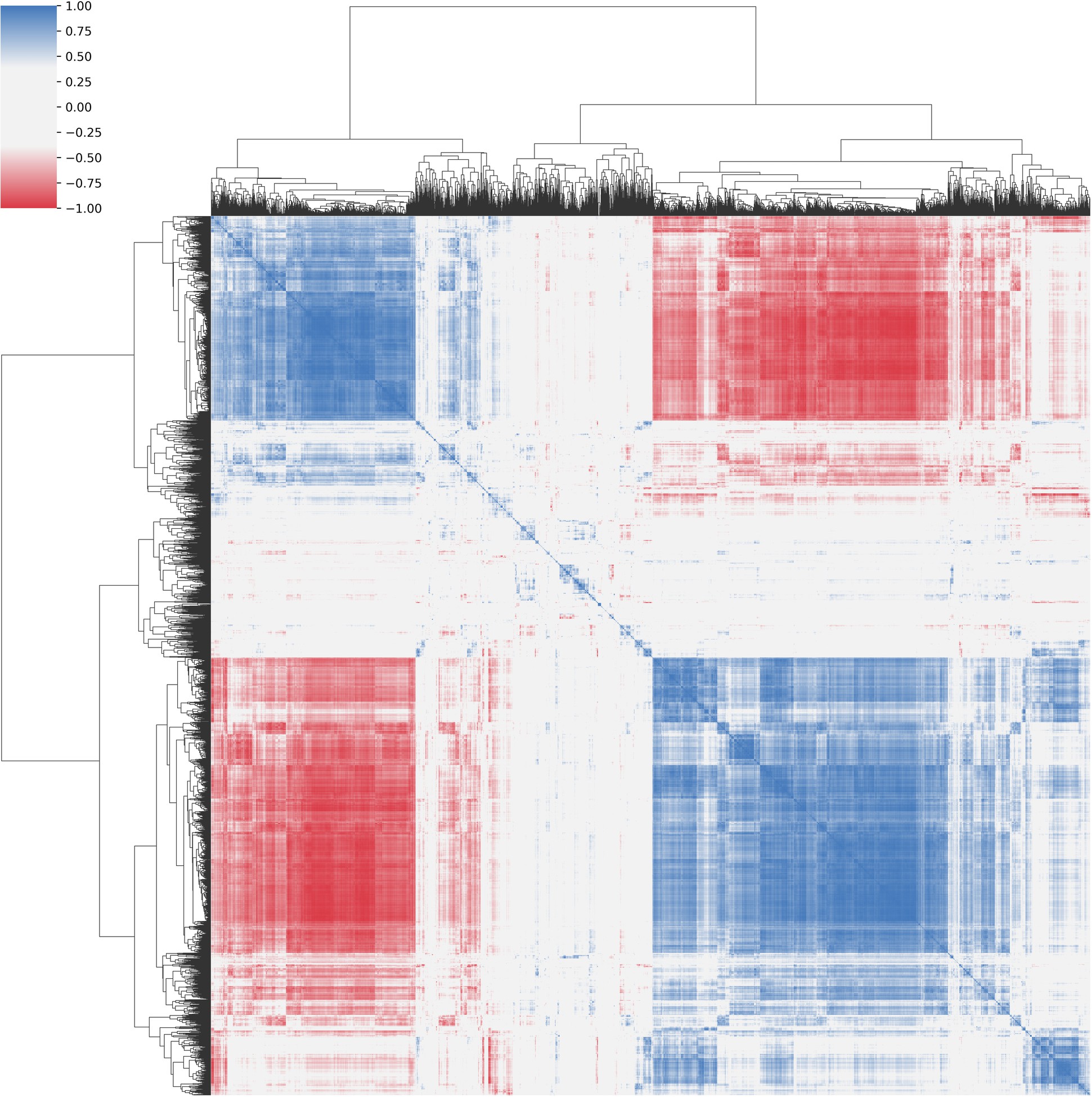


Supplementary Figure 5: Visualization of feature correlations through a heat map. The heat map exhibits symmetry about the diagonal. A value of -1 (deep blue) indicates a complete negative linear correlation, 0 (white) denotes no correlation, and +1 (deep red) signifies a complete positive correlation.
