# Supplementary material for: MRI-based radiomic features of the urinary bladder wall identify patients with moderate-to-severe international prostate symptom score
Source: World J Urol. 2024 Jun 13;42(1):375. doi: 10.1007/s00345-024-05081-3 (PMC11176201; doi:10.1007/s00345-024-05081-3)
Supplement: Supplementary file 9 — Supplementary Material 9 [file 345_2024_5081_MOESM9_ESM.docx]

Mean ROC curve with variability

Mean ROC (AUC = 0.87 *±* 0.09)

*±* 1 std. dev.

Chance level (AUC=0.5)

1*.*0

0*.*8

0*.*6

True Positive Rate

0*.*4

0*.*2

0*.*0

0*.*0 0*.*2 0*.*4 0*.*6 0*.*8 1*.*0

False Positive Rate

Supplementary Figure 6

: Mean receiver operating characteristic (ROC) curve with variability.
