# Supplementary material for: MRI-based radiomic features of the urinary bladder wall identify patients with moderate-to-severe international prostate symptom score
Source: World J Urol. 2024 Jun 13;42(1):375. doi: 10.1007/s00345-024-05081-3 (PMC11176201; doi:10.1007/s00345-024-05081-3)
Supplement: Supplementary file 10 — Supplementary Material 10 [file 345_2024_5081_MOESM10_ESM.docx]

Table 6: Optimal subset of features.

Feature

1: GLCM maximum a360 d1 b10 w1 f2 2: GLCM kurtosis a360 d3 b10 w3 f1

3: GLCM 4TH moment a180 d1 b5 w1 f2 4: GLCM kurtosis a180 d1 b10 w3 f1
