# Supplementary material for: MRI-based radiomic features of the urinary bladder wall identify patients with moderate-to-severe international prostate symptom score
Source: World J Urol. 2024 Jun 13;42(1):375. doi: 10.1007/s00345-024-05081-3 (PMC11176201; doi:10.1007/s00345-024-05081-3)
Supplement: Supplementary file 11 — Supplementary Material 11 [file 345_2024_5081_MOESM11_ESM.docx]

Table 7: Performance of the optimal subset of features.

| Metric | Value |
| --- | --- |
| Accuracy | 0.8056 |
| Balanced accuracy | 0.8095 |
| Recall | 0.8103 |
| Precision | 0.7750 |
| F1 | 0.7724 |
| ROC AUC | 0.8703 |
| Sensitivity | 0.8103 |
| Specificity | 0.8086 |
