# Supplementary material for: MRI-based radiomic features of the urinary bladder wall identify patients with moderate-to-severe international prostate symptom score
Source: World J Urol. 2024 Jun 13;42(1):375. doi: 10.1007/s00345-024-05081-3 (PMC11176201; doi:10.1007/s00345-024-05081-3)
Supplement: Supplementary file 12 — Supplementary Material 12 [file 345_2024_5081_MOESM12_ESM.docx]

Table 8: Performance of different subsets of features.

| Subset of features | Accuracy | Balanced accuracy |
| --- | --- | --- |
| Optimal | 0.8056 | 0.8095 |
| Naive | 0.5747 | 0.5000 |
| Only age | 0.6138 | 0.5733 |
| Only BMI | 0.5511 | 0.5127 |
| Only prostate size | 0.6687 | 0.6389 |
| Age, BMI and prostate size | 0.6650 | 0.6343 |
