# Supplementary material for: MRI-based radiomic features of the urinary bladder wall identify patients with moderate-to-severe international prostate symptom score
Source: World J Urol. 2024 Jun 13;42(1):375. doi: 10.1007/s00345-024-05081-3 (PMC11176201; doi:10.1007/s00345-024-05081-3)
Supplement: Supplementary file 13 — Supplementary Material 13 [file 345_2024_5081_MOESM13_ESM.docx]

Patients who had mpMRI images (n=140)

Excluded due to incomplete clinical data (n = 35)

Excluded as MRI didn't pass quality control step (n=18)

Patients included in analysis (n = 87)

Supplementary Figure 1: Consort Diagram: A Visual Representation of Study Participant Flow and Analysis
