# Supplementary material for: MRI-based radiomic features of the urinary bladder wall identify patients with moderate-to-severe international prostate symptom score
Source: World J Urol. 2024 Jun 13;42(1):375. doi: 10.1007/s00345-024-05081-3 (PMC11176201; doi:10.1007/s00345-024-05081-3)
Supplement: Supplementary file 14 — Supplementary Material 14 [file 345_2024_5081_MOESM14_ESM.docx]

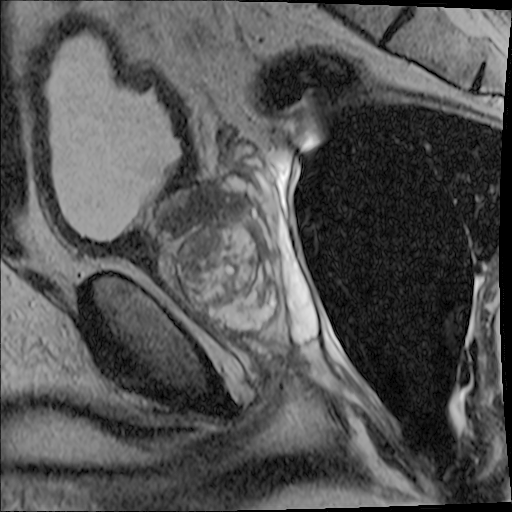

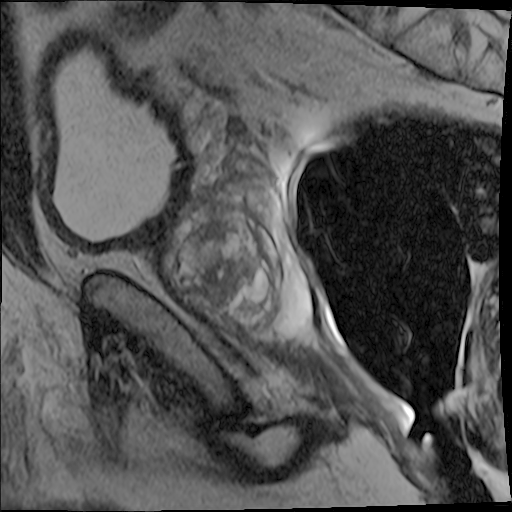

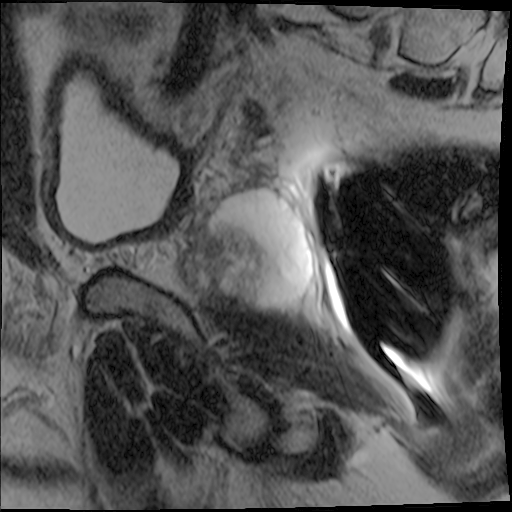

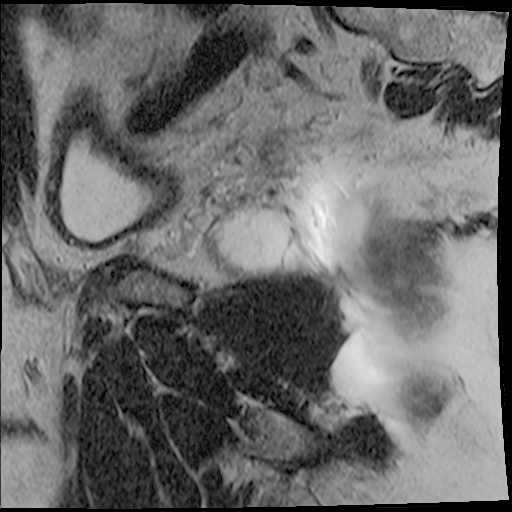


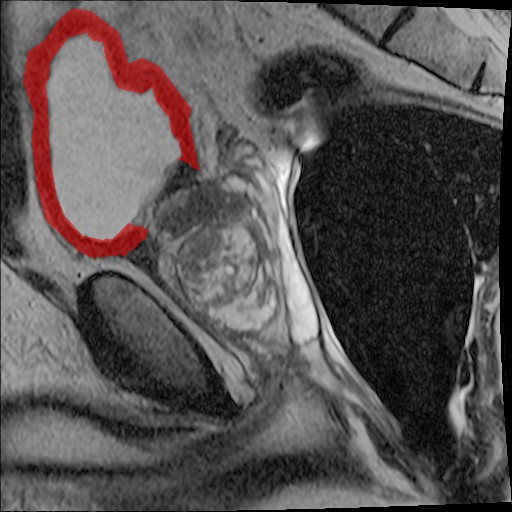

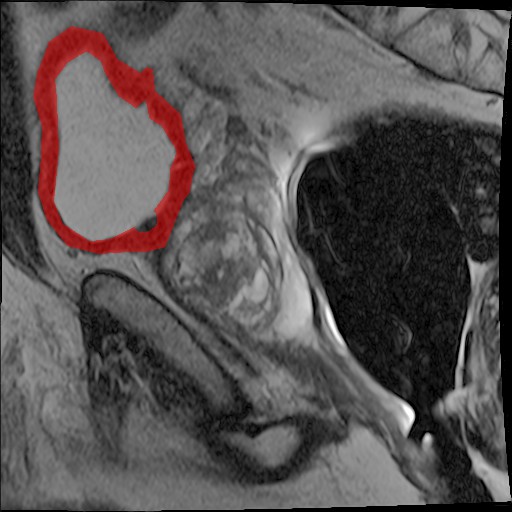

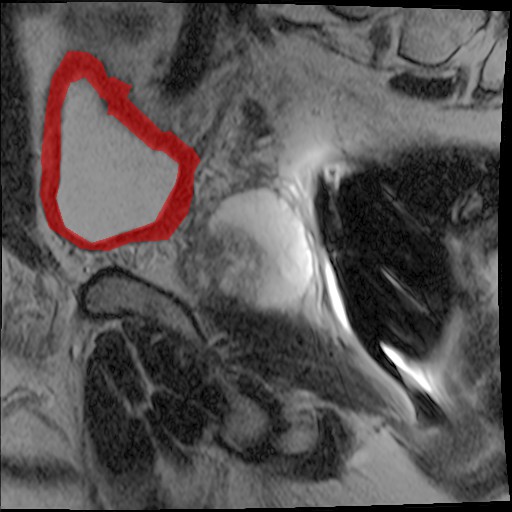

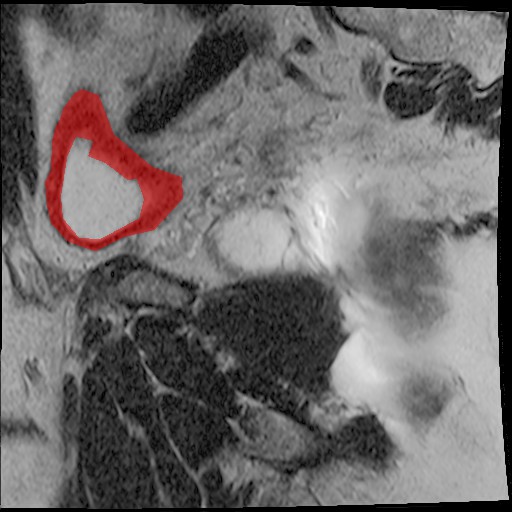


Supplementary Figure 2: Visual appearance of the bladder wall region in representative sagittal T2-weighted MRI images (top row) and of the delineated mask (bottom row) for the region of interest.
