# Supplementary figures and images for: MRI-based radiomic features of the urinary bladder wall identify patients with moderate-to-severe international prostate symptom score
Source: World J Urol. 2024 Jun 13;42(1):375. doi: 10.1007/s00345-024-05081-3 (PMC11176201; doi:10.1007/s00345-024-05081-3)

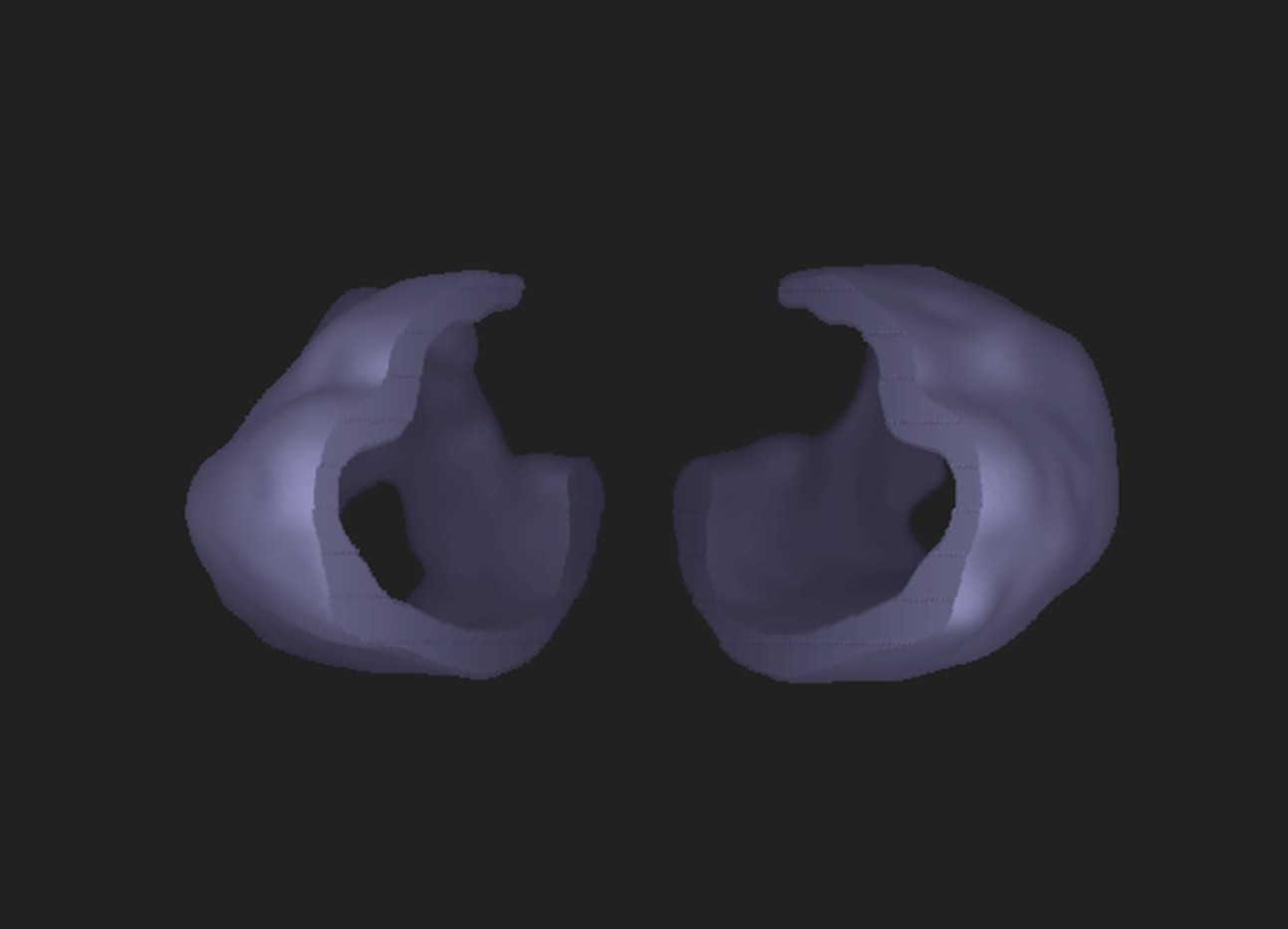


Supplementary Figure 3: 3D representation of bladder wall shell

Supplement: Supplementary file 15 — Supplementary Material 15 [file 345_2024_5081_MOESM15_ESM.docx]
